# Identification and Management of Paroxysmal Sympathetic Hyperactivity After Traumatic Brain Injury

**DOI:** 10.3389/fneur.2020.00081

**Published:** 2020-02-25

**Authors:** Rui-Zhe Zheng, Zhong-Qi Lei, Run-Ze Yang, Guo-Hui Huang, Guang-Ming Zhang

**Affiliations:** ^1^Department of Anesthesiology, Tongren Hospital, Shanghai Jiao Tong University School of Medicine, Shanghai, China; ^2^Department of Neurosurgery, The 901th Hospital of the Joint Logistics Support Force of PLA, Anhui, China; ^3^Department of Clinic of Spine Center, Xinhua Hospital, Shanghai Jiao Tong University School of Medicine, Shanghai, China; ^4^Department of Otolaryngology-Head and Neck Surgery, Ninth People's Hospital, Shanghai Jiao Tong University School of Medicine, Shanghai, China; ^5^Department of Neurosurgery, Shanghai Tenth People's Hospital, Tongji University School of Medicine, Shanghai, China

**Keywords:** paroxysmal sympathetic hyperactivity, traumatic brain injury, pathophysiology, clinical features, identification and management

## Abstract

Paroxysmal sympathetic hyperactivity (PSH) has predominantly been described after traumatic brain injury (TBI), which is associated with hyperthermia, hypertension, tachycardia, tachypnea, diaphoresis, dystonia (hypertonia or spasticity), and even motor features such as extensor/flexion posturing. Despite the pathophysiology of PSH not being completely understood, most researchers gradually agree that PSH is driven by the loss of the inhibition of excitation in the sympathetic nervous system without parasympathetic involvement. Recently, advances in the clinical and diagnostic features of PSH in TBI patients have reached a broad clinical consensus in many neurology departments. These advances should provide a more unanimous foundation for the systematic research on this clinical syndrome and its clear management. Clinically, a great deal of attention has been paid to the definition and diagnostic criteria, epidemiology and pathophysiology, symptomatic treatment, and prevention and control of secondary brain injury of PSH in TBI patients. Potential benefits of treatment for PSH may result from the three main goals: eliminating predisposing causes, mitigating excessive sympathetic outflow, and supportive therapy. However, individual pathophysiological differences, therapeutic responses and outcomes, and precision medicine approaches to PSH management are varied and inconsistent between studies. Further, many potential therapeutic drugs might suppress manifestations of PSH in the process of TBI treatment. The purpose of this review is to present current and comprehensive studies of the identification of PSH after TBI in the early stage and provide a framework for symptomatic management of TBI patients with PSH.

## Introduction

There is a subgroup of patients with simultaneously paroxysmal transient increases in sympathetic activity involved in heart rate, blood pressure, respiratory rate, temperature, sweating, and posturing activity, which may also persist over time, and are associated with worse outcomes (1). The unifying term for these syndromes—paroxysmal sympathetic hyperactivity (PSH)—which is caused by the dysregulation of the sympathetic nervous systems, was first recommended as a unifying term in 2010 (2, 3). Later, an unambiguous definition and diagnostic criterion for the syndrome was proposed by an expert group in 2014. Although the outbreaks of PSH were traditionally described in severe acquired brain injury (ABI) patients [e.g., traumatic brain injury (TBI), anoxic brain injury, stroke, tumors, infections, or unspecified causes], the prevalence of PSH of 33% after TBI compared with 6% after other causes suggests that the dominant underlying cause in PSH is TBI (4). In addition, in the past decade, about 80% of PSH cases have been reported to occur after TBI (5, 6). Its wide incidence rates reported ranging from 8 to 33% of PSH reveal the underlying discrepancy of current diagnostic and admission criteria as well as ignorance on disease identification (7).

There are about 8–10% of TBI survivors affected by this complication (8, 9). Previous studies have found that PSH was not an independent predictor for the increased morbidity or poor clinical outcome (2, 6). However, findings from other studies have suggested that a diagnosis of PSH in TBI patients was associated with longer hospitalization periods—approximately added at least 14 days—and worse clinical outcome had significantly lower motor scores and worse Glasgow Outcome Scale scores. The cause of increased mortality of PSH in severe TBI patients may result from those who did not respond to treatments rather than the complication itself, which leads to a prolonged duration of this complication, resulting in metabolic disorders or malnutrition and the deterioration of neurological condition occurring eventually (10, 11). However, a most recent case–control study, which was not consistent with regard to the effects of outcomes, revealed that the occurrence of PSH symptoms was not associated with more complications and higher mortality (12). Because of a lack of unified management process of PSH after TBI, between-study differences may ultimately emerge (3). Notwithstanding these uncertainties, the general impression of clinicians was that patients with severe PSH symptoms were more liable to suffer from poorer neurological outcomes.

Although the natural course between autonomic dysfunction and outcome of patients after TBI is not clearly understood, the objective quantification of such complications seems to be associated with global patient outcome (13). Given that septicemia, seizures, hydrocephalus, hypoxia, and other serious diseases invariably have overlapping manifestations with PSH, under-recognition and misdiagnosis occur frequently in clinical practice (7, 14, 15). For example, the manifestations of tachypnea and hyperthermia in PSH patients may empirically lead to a misdiagnosis of pulmonary embolism, the hyperthermia may mislead to a diagnosis of septicemia, and the posturing may mislead to an epileptic seizure. In addition, early identification of this condition is further hindered by the absence of a clear understanding of the pathophysiology of PSH, though the current consensus is that autonomic hyperactivity only concerns the sympathetic nervous system (3, 4, 16, 17). Currently, the Paroxysmal Sympathetic Hyperactivity-Assessment Measure (PSH-AM) scale consists of two separate constructs: (1) the clinical feature scale (CFS), to identify the intensity of cardinal features, and (2) the diagnosis likelihood tool (DLT), to evaluate the likelihood of the presence of PSH, and is by far the best diagnosis tool for PSH in TBI patients (1, 3). PSH-AM should contribute to the diagnostic criterion, enabling more systematic research on the identification and management of PSH.

It is generally accepted that poor long-term outcome in PSH patients is associated with a low level of consciousness recovery, and early appearance and long duration of severe dysautonomic symptoms (18). Conventional treatments for PSH include analgesia, sedation, and muscle relaxation. However, treatment-related events such as prolonged respiratory support in the intensive care unit (ICU), and a delay in early neurological rehabilitation, may lead to the deterioration of neurological function. Recently, a variety of new therapeutic strategies, acting on different functional mechanisms, have assisted the process of PSH treatment (18). Unfortunately, individual differences in pathophysiological processes might hinder the establishment of precise therapeutic strategies (19). Even so, accurate identification of the dominant symptom and the formulation of symptomatic treatment provide a foundation for the effective treatment of PSH until further screening can be carried out.

The purpose of this review is to provide an overview of the pathophysiological mechanisms, clinical features, and identification methods, and discuss the substantial commonalities of therapeutic options for PSH. We also present an algorithm for the identification and management of PSH after TBI.

## Pathophysiology

**Figure 1:**
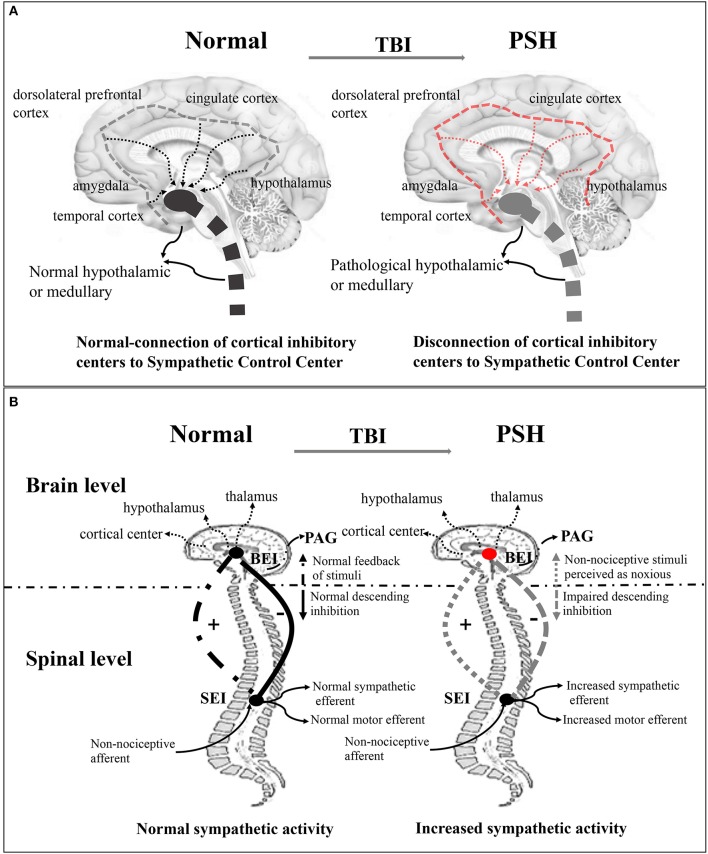
Disconnection theory and EIR model of the pathogenesis of PSH of the pathogenesis of PSH. **(A)** Disconnection theory: Left-sided, the normal connection of cortical inhibitory center (insula and cingulate cortex) to sympathetic control center (hypothalamic, diencephalic, and brainstem centers) in the normal brain. Right-sided, the disconnection of cortical inhibitory centers to sympathetic control center in the TBI brain. **(B)** EIR model: Left-sided, in the brain level, the normal brainstem center (cortical and subcortical center, hypothalamic, and thalamic) inputs modulate activity and then provide inhibitory drive to spinal reflex area; in the spinal level, the normal spinal centers give upward feedback of sensory and perception stimulus for one thing, and output sympathetic and motor efferents for another, thereby maintaining balance between inhibitory and excitatory interneuron activity. Right-sided, the disconnection of descending inhibition produces the excitation of feedback loop where non-noxious stimulus is potentially perceived as noxious stimulus. EIR model, Excitatory Inhibitory Ratio model; TBI, traumatic brain injury; PSH, paroxysmal sympathetic hyperactivity.

Several theories exist regarding the pathophysiology of PSH syndrome, although early epileptogenic theories have been abandoned for lack of empirical evidence (3). Existing disconnection theory (Figure 1A) indicates that severe paroxysmal activity is associated with the notion of diencephalon–upper brainstem release (3, 20, 21). The underlying mechanisms have been described more precisely, as the study of the autonomic nervous system in the brain has furthered. These mechanisms include the generation of a sympathetic tone in the brainstem, hypothalamus, and the spinal cord, and the inhibition of sympathetic discharge in cortical structures, such as the hippocampus, amygdala, insular cortex, cingulate cortex, middle temporal cortex, and dorsolateral prefrontal cortex (22, 23). Subsequently, a more detailed study showed that disconnection of one or more cerebral centers or disturbances in cortical and subcortical regions caused by focal or diffuse injuries were responsible for autonomic dysfunction (21). In this theory, the anterior hypothalamus, or medulla, is regarded as the primary region implicated in central sympathetic nervous system activation (21, 24, 25). Despite existing evidence supporting theories of disconnection of cerebral inhibitory pathways from excitatory centers, they are insufficient in explaining all of the symptoms observed in PSH patients (21). Even so, in a non-TBI case, excessive inflammatory conditions in the cerebral cortex and subcortical white matter, rather than in the brainstem or other lower regions associated with PSH, were reported, supporting this theory (26). Currently, a widely accepted theory involves the excitatory/inhibitory ratio (EIR) (Figure 1B), which describes PSH as a two-stage pathological process. First, excitation originates from the disconnection of descending inhibitory pathways, and second, the paroxysm is halted by the recovery of inhibitory factors (3, 4, 9, 21, 27). The EIR model represents a mechanism where motor and sympathetic overactivity originate from the spinal and/or central level, and in TBI, inhibition of descending and afferent non-noxious feedback is impaired (21, 27, 28). This theory provokes the pathologically reinterpretation that patients' differential response to slight-noxious or non-noxious stimuli is caused by individual allodynic tendency to the reaction (27). It also assumes that the paroxysm of sympathetic symptoms may be a response to structural or functional impairment of the midbrain in TBI patients (3). Furthermore, this model explains why those with less brainstem involvement have a shorter duration of paroxysm and a much easier recovery of upper-spinal inhibition.

Although the anatomical basis of the pathogenesis of PSH is still undefined, research has indicated that specific TBI damage characteristics will increase in occurrence. The presence of focal parenchymal lesions within the brain increases the likelihood of developing PSH (29). More detailed characterization of structural lesions has been gained by neuroimaging technologies (9, 30). Those patients with deeper brain injuries in the periventricular white matter, corpus callosum, diencephalon, or brainstem are more likely to develop PSH than those with cortical and subcortical injuries (31). The emergence of PSH is often associated with scattered lesions or diffuse axonal injury, particularly with disturbance in white matter of the corpus callosum and posterior limb of the internal capsule (32). Unfortunately, current research has been unable to evaluate the location of the injury lesions or lesion lateralization in the development of PSH, for several reasons. First, severe TBI usually causes diffuse injury and therefore lacks the specific driving lesions for PSH development; second, there is absence of large-scale standardized clinical imaging data; third, it is difficult to isolate the contribution of the complex symptoms of PSH from the overall burden in TBI patients (3, 21). Taken together, the contributions of specific cortical and gray matter, or their relationship, should be a focus for research in the future.

Recently, neuroendocrine regulation disturbances observed in the maladaptive response provide new insights for the pathophysiology of PSH (33). In the neurotransmitter system, paroxysm derives from uncontrollable adrenergic outflow resulting in increased circulating catecholamine (34, 35). Research suggests that levels of adrenocorticotropic hormone (ACTH), epinephrine (E), norepinephrine (NE), and dopamine (D) significantly increase during paroxysm, while NE and D decrease, rather than ACTH and E, during the intermittent period (4, 17, 36). This is because NE and D arise from increased excitability of the sympathetic nervous system, whereas E is almost exclusively derived from the adrenal medulla (37). In general, there is about a 2- or 3-fold increase in catecholamine and an ~40% increase in adrenocortical hormones in serum (17, 38). Changes in the neurotransmitter system, driven by stimuli, highlight the importance of considering the triggering event in pathogenetic research. To summarize, the triggering of paroxysm, a sudden exaggerated response originating in the afferent stimulation of the sympathetic nervous system, is responsible for the core pathophysiological features (38, 39).

## Clinical Features

The primary clinical feature of PSH is simultaneous, paroxysmal transient increases in sympathetic, as well as motor activity (1). Although consensus on the isolated symptoms of this complication consists of six core sympathetic and motor features (tachycardia, tachypnea, hypertension, hyperthermia, hyperhidrosis, and posturing), PSH is a complex syndrome that shows individual differences across a spectrum of clinical symptoms (1, 9, 16). In fact, few patients present with all symptoms, while the vast majority of patients present with a single combination or various combinations of core symptoms (11). This is partly because individual differences or some symptoms are masked by the TBI therapeutic process (e.g., analgesic and sedative) (3). A previous study reported that tachycardia was almost uniformly seen in all patients, whereas the rest of the core features were relatively rare (15). The presence of these symptoms was related to some unexpected events, such as higher overall mortality, longer recovery time, higher risk of infection, and other worse outcomes (7). Meanwhile, some unforeseen comorbidities, such as cardiac involvement, weight loss, heterotopic ossification, and immunodepression, invariably accompany the core symptoms of PSH (40–43). For instance, resting energy expenditure in the paroxysmal state was three times higher than baseline, and weight loss was 25–29% in patients when entering the rehabilitation stage (3). Recent studies have suggested that three of the core symptoms, hypertension, diaphoresis, and dystonia, can be considered as predictive signs of pediatric PSH, relative to adults (44, 45). In all six-core symptoms, motor symptoms (e.g., dystonia or posturing) and long-lasting (recurring from early to chronic stages) symptoms are often difficult to identify (15, 27, 46).

Clinically, up to 72% of PSH patients with the above symptoms are caused by the unavoidable non-noxious stimuli (7, 9). Some TBI or treatment-related stimuli, such as pain, suction, passive motion, or postural changes, are regarded as the predisposing factors of PSH (2, 3). When referring to the peak time of onset, it was not known whether there was any incubation period of PSH. It is always occurring in the early stage of TBI, especially within 1 week after TBI, and the incidence rate will decrease with the recovery of brain injury (6, 8). Given that the paroxysmal symptoms neither appear suddenly nor cease abruptly, post-traumatic time is not an optimizing predictor for PSH diagnosis (47). Further, the duration of PSH is variable. The majority of patients will recover in a few weeks, while fewer severe cases remain in a low-response state of rehabilitation for several weeks to months, even more than 1 year after injury (6, 7, 9). With the time window of each episode being within a few minutes to 2 h, duration is influenced by individual differences and management measures (9, 11, 16, 48). A previous study of PSH in the ICU suggested that the average episode duration was about half an hour (9). With reference to the daily self-limitation of PSH, researchers found that the average frequency of episodes is about 5.8 times a day, through collection of qualitative data from different literature (9, 38). Episode severity is reduced with the duration of disease, and the natural course (from initial injury to the asymptomatic phase) of PSH is about 2 weeks in general (6, 49). To our knowledge, several features, such as sweating (the commonest), tachycardia, and posturing will continue to the rehabilitation stage of TBI. Moreover, in all core secondary symptoms of PSH, the second damages caused by tachycardia, tachypnea, and hypertension are often more severe than the rest of the core symptoms (47).

In the past, long-term clinical observations have revealed that PSH is more prevalent in men than in women (8, 9, 50). Moreover, younger age was significantly associated with the development of PSH in adults (9, 51). The most recent research indicates that older age, among children aged from 1 month to 18 years, was associated with an increased risk for developing PSH (44, 52). From this, we can presume that PSH most likely occurs in young men, although there is no consensus as to the mean age (2, 16, 44). When it comes to the relationship between PSH and the severity of TBI, previous studies suggest that PSH always appears in patients with a lower Glasgow Coma Score (GCS), especially those with lower than 8 points, that is, patients with severe TBI. Further diagnostically useful findings suggest that the GCS decreased during episodes and relatively increased in the remission phase of PSH (9, 52, 53). Researchers also use the clinical features severity scale (CFSS) to quantify clinical features and show that PSH is more likely to develop in patients with temperatures over 38.0°C, or rising, during the first 24–72 h post-injury (54). In addition, some unusual features deserve attention: hot or painful joints in PSH may be caused by heterotopic ossification, PSH is associated with prolonged tracheostomy weaning in severe TBI, and the use of tracheostomy might be independently associated with an increased incidence of PSH (41, 55, 56).

## Identification of PSH

**Figure 2:**
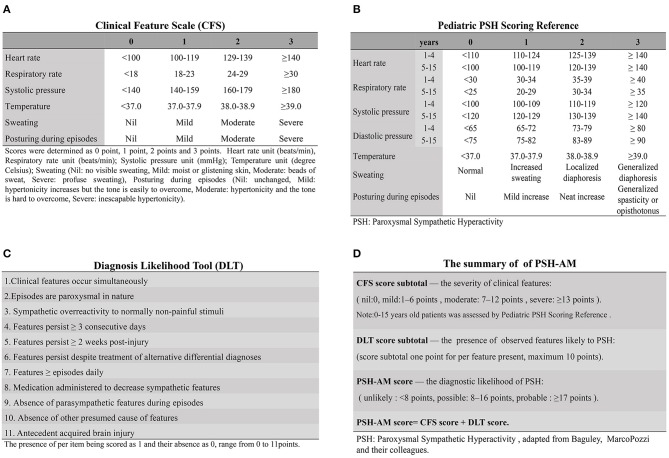
The PSH-AM tool and clinical application. The PSH-AM tool contains two constructs: **(A)** the clinical feature scale (CFS), which assesses the intensity of the six-core features identified to PSH; and (**B)** the diagnosis likelihood tool (DLT), which identifies the presence of observed features, thereby estimating the likelihood of those that are due to PSH. **(C)** Clinical Scales for PSH in Pediatric Patients are able to provide a more fine-grained estimation, where the parameters could be evaluated separately and then yield more information. **(D)** Clinical application of PSH-AM Tool: the total PSH-AM scores (combined with the CFS and DLT subtotal scores) give an estimate of the probability of a diagnosis of PSH [adapted from Baguley, MarcoPozzi, and their colleagues (3), by permission of Mary Ann Liebert, Inc.] PSH-AM tool, Paroxysmal Sympathetic Hyperactivity Assessment Measure; PSH, paroxysmal sympathetic hyperactivity.

Symptom-based findings are used for the early identification of PSH in TBI patients. In 2014, the PSH assessment measure (PSH-AM) tool (Figures 2A,B), a clinical scoring system used for probabilistic diagnosis, was proposed by an expert consensus group (1). In order to maintain diagnostic consistency, 11 pathognomonic signs were retained, while five previously reviewed features were excluded from this tool (1, 3). There are two components in PSH-AM; the DLT for measuring the presence of compatible features of PSH, and the CFS for assessing the severity of excitement of sympathetic nerves, as well as motor activity, on a scale of 0–3. A DLT score of 1 is recorded when the diagnostic feature, as described above, is present. On this scale, the higher the scores, the greater justification for the establishment of diagnosis. Combined scores (Figure 2D) indicate the diagnostic likelihood of PSH, as unlikely (scores <8), possible (scores 8 to 16), or probable (scores ≥17) (1).

Previous and recent cases provide evidence that PSH-AM can not only serve as reliable diagnostic criteria, but also stratify the severity of PSH (10, 57). The tool is able to dynamically monitor the evolution of individual PSH patients' clinical status, and is also valuable in diagnosing PSH in different types of brain trauma [e.g., diffuse axonal injury; (57, 58)]. Given that further assessment and inspection due to persistent symptoms is necessary in the treatment of patients with TBI, PSH-AM can help to avoid misdiagnosis, enhance diagnostic efficiency, save time, and reduce economic costs. The prospective predictive value of the PSH-AM tool has been confirmed in sensitivity/specificity analysis, showing higher sensitivity, though limited specificity (10). A recent retrospective analysis suggested that the incidence of diagnosed PSH cases in TBI patients was reduced from 32 to 18%, compared with previous studies, using this tool (59). However, some limitations affect the identification of PSH in TBI patients. First, the isolated feature of PSH may be hidden in many TBI-related complications such as seizures, sepsis, hypoxia, hypoglycemia, and traumatic pain. Second, some diagnostic criteria are only suitable for use after special clinical features first appear. Third, misdiagnoses are common despite a rigorous diagnostic approach (3, 9). Furthermore, fresh arguments hold that the baseline parameters of PSH should be based on age-matched specifications, and the consensus of PSH cannot be generalized (60). For clinical experiences, for pediatric patients with greater variability of heart rate or blood pressure, the specificity of PSH-AM is less than that for adults (44, 45). To remedy this, a pediatric scoring system (Figure 2C) was adopted in a pediatric rehabilitation center. The significant differences to CFS are the specificity of the evaluation index and method among pediatric patients (47, 61). Unfortunately, multi-center assessment of the application and popularization of this method is lacking.

Nevertheless, it is important for clinicians to avoid rejecting a diagnosis, due to the absence of a certain feature or inconsistencies with the proposed diagnostic criteria (15, 62). Currently, many reports have presented empirical evidence for the early identification of PSH after TBI. Although PSH cannot be diagnosed accurately by laboratory examination, a diagnosis of exclusion should rule out the following: infectious disease (e.g., pneumonia or sepsis), drug-induced disease (e.g., fever or neuroleptic malignant syndrome), rhabdomyolysis, dehydration, seizures, pulmonary embolism, or deep vein thrombosis (43). In TBI patients, negative microbial cultivation of blood, cerebrospinal fluid, airway secretion, or urine provides clues for exclusion. In addition, normal electroencephalograms (EEGs) in PSH patients can help to exclude epilepsy and other nervous system diseases (7, 39, 63, 64). In brief, those examinations could improve the efficiency of diagnosis before the preliminary symptoms and validation have occurred (64).

Furthermore, some clues have emerged through the use of imaging techniques, in predicting the onset of PSH in TBI patients. First, a previous study suggests that the presence of focal lesions on computer tomography (CT) images during the first 48 h was associated with a greater probability of PSH crises as compared with the presence of diffuse lesions or a normal CT in TBI patients (29). Subsequent magnetic resonance imaging (MRI) suggests that PSH is more likely to be found in patients with deep structural as well as diffuse brain damage, while grouping lesions into three different classifications (cortical and subcortical white matter, corpus callosum or diencephalon, and dorsolateral aspect of the midbrain and upper pons) (31). Diffusion tensor imaging (DTI) suggests that low fractional anisotropy (FA) values (a measure of the disconnectivity of white matter) in the right-side posterior of the internal capsule and the splenium or corpus callosum have a significant correlation with the development of PSH (32). Because the pathology of PSH is unclear, these findings are unable to precisely identify the neuroanatomical characterization bias of PSH and thus cannot provide a confirmed diagnosis.

## Treatment Progress

Traditional views hold that obstacles to the development of PSH treatment are the following: (1) insufficient understanding of brain regions, (2) no definite relationship between neurotransmitters or hormones and clinical symptoms, (3) lack of standardized measures to assess the curative effect, and (4) insufficient evidence from clinical trials regarding the benefits of intervention for long-term outcomes (2, 3, 10, 65, 66). However, significant progress has been made in the management of this complication with the goals of avoiding the triggering event, relieving excessive sympathetic nerve activity, and alleviating adverse effects (3, 43, 65). Prior to treatment, the first step is discerning the necessity and urgency of which symptoms need to be priority processed (43). For instance, the key step in treatment of PSH patients with hyperhidrosis is sufficient fluid replacement, rather than control of the sympathetic outbreak, because dehydration will reduce consciousness, and either positive pharmacotherapy or external cooling is critical for hyperthermia, as fever is inherently harmful (19). Then, different types of core symptoms that are involved in the different phase of PSH should be determined. How to apply pharmacotherapy and supplementary treatment in the interval phase and whether to adopt supportive therapy during the rehabilitation stage are the most common problems faced by clinicians, by far (3, 43). Previous experience holds that there is a sympathetic positive feedback loop in patients with long-term duration that is impossible to disrupt, making treatment all the more difficult (67). Last, it is important to consider the optimal choices in terms of timing, route, and cycle of treatment (65, 68). Here, we classify treatment methods into two main types, pharmacologic and non-pharmacologic.

### Pharmacological Treatment

Clinicians realize that pharmacological interventions should depend on the comprehensive analysis of different types of symptoms and individuals' differences, and a range of medicines have been used in PSH treatment (3, 65, 69). In clinical practice, most patients require treatment with multiple drugs with potential complementarity both to target different syndromes and to prevent or treat paroxysms. At this point, individual drugs and drug combinations are typically chosen on the basis of clinical experience. In Table 1, we present the current pharmacologic treatments with their advantages and disadvantages used in the prevention or treatment of PSH.

**Table 1:** The recommended class of medications for the treatment and prevention of PSH.

**Opioids**		
Morphine	Mechanism	μ opioid receptor agonist (brain and spinal cord) modulates the central pathways responsible for autonomic dysregulation (4, 19).
	Methods	1–10 mg intravenously in the treatment of PSH, higher doses (up to 20 mg) in severe cases, intravenous infusion for prevention.
	Target features	Most features (particularly hypertension, tachycardia, and allodynia) (3, 19, 70).
	Advantages	Therapeutic effect is rapid and reliable, the most effective drug to relieve episodes of severe PSH, can be used until the rehabilitation stage (19, 53, 65, 69, 71).
	Disadvantages	Dose-dependent (requires relatively large doses in some case), withdrawal symptoms may occur after prolonged use, and the major side effects are respiratory depression, sedation, or hypotension (19).
Fentanyl	Mechanism	μ opioid receptor agonist (brain and spinal cord).
	Methods	10–30 mcg/h, fentanyl propenamide patch, tapered gradually after 1 week (72).
	Target features	Most features (particularly hypertension, tachycardia, and allodynia).
	Advantages	Reusable (72).
	Disadvantages	Have not been described.
**Intravenous anesthetics**		
Propofol	Mechanism	GABA_A_ receptors in the brain.
	Methods	Prevention: intravenous infusion <4 mg/kg per h; treatment: 10–20 mg intravenous injection.
	Target features	Most features, refractory symptoms (66).
	Advantages	Can be used in the acute phase.
	Disadvantages	Needs respiratory support with mechanical ventilation.
**β-Adrenergic blockers**		
Propranolol	Mechanism	Non-selective β blockers (central, cardiac, and peripheral), effective in reducing the role of circulating catecholamine and thus lowering the resting metabolic rate (3, 17, 19, 73).
	Methods	Dosage should be specific (a high dose may cause hypotension or bradycardia), 20–60 mg per 4–6 h (oral or intestinal) (3, 4, 19, 47, 65).
	Target features	Hypertension, tachycardia, diaphoresis, and perhaps help with dystonia (3, 19, 66).
	Advantages	The most frequently used, reducing the incidence of secondary injury or mortality rate, better than most other members of the family in lipophilicity and penetration of the blood-brain barrier, normalizing blood pressure, further lowering heart rate and improving myocardial function (19, 47, 74–76).
	Disadvantages	Mainly ameliorates the consequences of the disorders rather than the central mechanisms responsible for the autonomic dysfunctions, and the major side effects are bradycardia, hypotension, arrhythmia, or hypoglycemia, and possible hypoglycemia in patients receiving insulin therapy (3, 19, 76).
Metoprolol	Mechanism	β_1_-blocker.
	Methods	Prevention: 100–200 mg per 8 h, oral.
	Target features	Hypertension, tachycardia.
	Advantages	The mainstream drug can be used for long-term administration (65).
	Disadvantages	β_1_ antagonism alone is not sufficient to suppress PSH, possibility for heart block (4, 77).
Labetalol	Mechanism	β_1_+β_2_ and α blocker (both central and peripheral) exert a stabilizing effect within the central nervous system through indirect inhibition of sympathetic activity (77).
	Methods	Prevention: 100–200 mg per 8 h, oral.
	Target features	Hypertension, tachycardia, and diaphoresis.
	Advantages	Leads to an observable decline in symptoms, can reduce peripheral vascular resistance, blood pressure, and coronary vascular resistance (77).
	Disadvantages	The major side effects are bradycardia, hypotension, arrhythmia, or hypoglycemia.
**α_2_-agonists**		
Clonidine	Mechanism	Presynaptic α_2_-receptor agonist (brain and spinal cord), effectively reduces catecholamine levels in circulating plasma, decreases the hypothalamus and ventrolateral medulla sympathetic outflow, and thus enhances brainstem sympathetic suppression (16, 65, 78).
	Methods	Prevention: 100 μg per 8–12 h (oral or intravenous infusion), <200 μg/day, can be used for epidural or intestinal administration.
	Target features	Mostly hypertension and tachycardia.
	Advantages	A wide range of administration, can be used in combination therapy.
	Disadvantages	Usefulness is limited, relatively ineffective for other symptoms, thus requires combination with agents with different mechanistic actions, treatment-related hypotension often observed in the therapeutic process; the major side effects are hypotension, bradycardia, sedation, withdrawal reaction (mostly in epidural administration), depression, and constipation (19, 79).
Dexmedetomidine	Mechanism	α_2_ agonist (brain and spinal cord), inhibits central sympathetic outflow without affecting sympathetic feedback, effective for sedation, and analgesia (66).
	Methods	Prevention and treatment: intravenous infusion, 0.2–0.7 μg/kg/h.
	Target features	Hypertension, tachycardia dystonia, pain, and anxiety (3, 66).
	Advantages	Widely used in the intensive care unit to alleviate pain and anxiety, maintain the stability of hemodynamics, with less respiratory depression without requirement for mechanical ventilation, easy to wake patients up to judge the consciousness state, can be used as a preventive drug for PSH in TBI patients (66, 80, 81).
	Disadvantages	The major side effects are hypotension, bradycardia, and sedation; intravenous injection is not a long-term solution (3).
**Benzodiazepines**		
Diazepam	Mechanism	GABA_A_ agonist (brain and spinal cord), increases the opening of chloride channels after benzodiazepine-induced inhibition of electrical activity (3).
	Methods	Treatment: 1–10 mg intravenous injection
	Target features	Agitation (first choice), hypertension, tachycardia, and dystonia and spasticity (65, 66, 74).
	Advantages	Has a good liposolubility.
	Disadvantages	Less effective than opiates, probably worsens neurological functioning; the major side effects are sedation, hypotension, and respiratory depression, carefully if without artificial airways (19).
Lorazepam	Mechanism	GABA_A_ agonists (brain and spinal cord).
	Methods	Treatment: 1–4 mg intravenous injection.
	Target features	Agitation, hypertension, tachycardia, and posturing.
	Advantages	Long duration.
	Disadvantages	The major side effects are sedation, hypotension, and respiratory depression, use carefully if without artificial airway.
Midazolam	Mechanism	GABA_A_ agonists (brain and spinal cord).
	Methods	Treatment: 1–2 mg intravenous injection.
	Target features	Agitation, hypertension, tachycardia, and posturing.
	Advantages	Rapid onset and short duration.
	Disadvantages	The major side effects are sedation, hypotension, and respiratory depression, use carefully if without artificial airway.
Clonazepam	Mechanism	GABA_A_ agonists (brain and spinal cord).
	Methods	Prevention: 0.5–8.0 mg/day, oral.
	Target features	Agitation, hypertension, tachycardia, and posturing.
	Advantages	Can be used for prevention, has good liposolubility.
	Disadvantages	The major side effects are sedation, hypotension, and respiratory depression.
**Neuromodulators**		
Bromocriptine	Mechanism	Synthetic dopamine agonist, the mechanism for the treatment of dysautonomia is unclear (66).
	Methods	Prevention: 1–25 mg per 12 h, oral, <40 mg/day.
	Target features	Hyperpyrexia and sweating (second-line drug) (3).
	Advantages	Effectiveness is enhanced in combination therapy, especially with morphine, and halts the persistent episodes (3, 19, 66, 82).
	Disadvantages	Uncontrolled hypertension and high-risk of seizure; major side effects are hypotension, confusion, dyskinesia, and nausea (3, 4, 19).
Gabapentin	Mechanism	GABA agonist derivative that acts on the α2δ presynaptic voltage-gated Ca^2+^ channels (brain and spinal cord) (3).
	Methods	Prevention: 100 mg per 8 h, 4,800 mg/day, oral.
	Target features	Spasticity, hyperpyrexia, and allodynia, reduces the frequency of paroxysm (19, 83).
	Advantages	Well-tolerated, applicable for the acute or recovery phase, long-term application (83).
	Disadvantages	Mild sedation (19).
Baclofen	Mechanism	GABA_B_ agonist, resulting in a primary effect at the dorsal horn of the spinal cord if administered intrathecally (83).
	Methods	Prevention: 5 mg per 8 h, 80 mg/day, oral; intrathecal injection (3, 18, 84).
	Target features	Spasticity (decreases the frequency and severity), dystonia, clonus, post-traumatic pain (66, 69, 85).
	Advantages	Intrathecal injection of baclofen (ITB) will facilitate reduction of or dispensing with oral baclofen or propranolol, and is useful in refractory patients; intra-ventricular baclofen seems to be a safer alternative choice than ITB (18, 51, 84, 86).
	Disadvantages	ITB is less effective than additional oral administration when used concomitantly, use is restricted to spinal cord injury patients, ITB is not popular (high risks of cerebrospinal fluid leakage and infection, mechanical problems with the catheter or pump, operation is difficult in patients with abnormal anatomy); major side effects are sedation and withdrawal syndrome (fever, rigidity, dystonia, or seizures) (3, 19, 69, 84, 85).
**Peripherally acting muscle relaxants**		
Dantrolene	Mechanism	Peripheral sarcolemma Ca^2+^ release blockers, produce muscle dissociation of excitation–contraction through interfering with calcium release from the sarcoplasmic reticulum (3, 19).
	Methods	Intravenous injection 0.5–2 mg/kg per 6–12 h, <10 mg/kg/day.
	Target features	Posturing and muscular spasms.
	Advantages	Significantly ameliorate malignant hyperthermia and particularly for severe dystonic posturing, can be combined with barbiturates, benzodiazepines, or opiates for refractory treatment (4, 19, 66).
	Disadvantages	Need to monitor liver function during use; the major side effect is hepatotoxicity respiratory depression (3, 19).

Unfortunately, as there is no medication that is especially effective for PSH, or which can eliminate a second occurrence, a brief historical overview will be presented, on the basis of subjective rather than objective evidence (3). For example, opioids and β-blockers are widely accepted medications; the first-choice medicines are most opioids, gabapentin, benzodiazepines, and central α-agonists or β-antagonists; bromocriptine, commonly used in drug combinations, is the second choice (2, 65, 69). Nevertheless, previous studies have suggested that drug combinations are more effective for symptom control among patients with PSH (3, 74). Moreover, recognizing episodes promptly, avoiding unnecessary therapies for alternative diagnoses, adjusting dosage, or switching to a different medication according to the progress of the disease can improve the curative effects (19). Recently, a retrospective study found that the initial severity of symptoms has no obvious significance in determining drug selection and thus overturns the hypothesis that medication may affect the progression of PSH (47). Optimizing the therapeutic effects and minimizing the side effects of these medications are the key goals of clinicians, in general, and choosing short-acting medications with the appropriate regimen, and avoiding over-administration, is critical for effective treatment.

Conventional therapies for PSH are oral or intravenous drugs, including sedative and analgesic drugs, muscle relaxants, and antiadrenergic drugs. However, recent research points out that these modes of administration are ineffective for treatment, in part because long-term sedation and analgesia will delay neurological rehabilitation and thus probably induce deterioration of neurological function. Since low tolerance and high dose-dependence of medication (e.g., baclofen) occurs in some severe cases, a switch to intrathecal injection is recommended, not only in treatment after initial treatment failure, but also makes other drugs dispensable (18, 66).

In view of the above understanding, there are three treatment approaches: symptom elimination, symptom prevention, and refractory treatment (66). With the characteristic of rapid onset and short half-life, medications applied for symptom elimination can immediately break down paroxysmal episodes. In the past, morphine and short-acting benzodiazepines were the drugs of choice, because of their efficacy in clinical practice. The optimum application periods of eliminative medications are the early stages of the paroxysmal period. Although which one should firstly be applied is uncertain, the therapeutic basis of such medications is the predominant symptom of PSH. In general, different goals of these medications are fever-reducing in hyperthermia, heart rate controlling in tachycardia, dynamic maintaining blood pressure, timely and proper sedation, and relieving spasticity or decreasing muscular tone (3, 19, 66). Symptom preventative medications are used for decreasing the frequency and intensity of PSH patients' symptoms. There are many drug categories, such as non-selective β-blockers, α2-agonists, gabapentin, baclofen, bromocriptine, and long-acting benzodiazepines, which have achieved great efficacy in clinical practice (66). Regarding refractory treatment, it must begin with the recognition that certain symptoms are insensitive to treatment and some symptoms (e.g., hyperthermia or hypertension) can lead to secondary injury. It is noteworthy that problems such as hyperpyrexia, posturing, and autonomic instability originate from neuroleptic malignant syndrome (NMS), which is caused by long-term use of chlorpromazine or haloperidol, and are common symptoms of PSH. Last, if intractable symptoms appear, continuous infusion of intravenous medications such as propofol, benzodiazepines, opioids, or dexmedetomidine can be offered, until they subside. Importantly, it is important that clinicians know that combination therapy may be necessary to prevent persistent outbreaks, and that preventative medications should firstly be considered for persistent symptoms and chronic symptoms that are difficult to control.

However, an entirely persuasive meta-analysis is not possible because of heterogeneity and poorly documented published data (3). There are several limitations of systematic pharmacologic studies: (1) the lack of advice on the distribution and metabolic effects of administration route, form, or dose, (2) few have concentrated on the prevention of PSH in TBI patients, and (3) results may not be generalizable due to heterogeneity of the sample population and the absence of adequate statistical power or long-term follow-up data after discharge (3, 19, 66, 80). In view of the above, the focus of ongoing clinical trials is to increase data reliability through multicenter studies, balancing the effects and side effects of the different medications, thus providing results that are useful in clinical practice (3, 4, 87).

### Non-pharmacological Treatment

Recent studies have recommended some non-pharmacological treatment methods for PSH (14). Before initial treatment, environmental modification is an important measure. Controlling room temperature to provide a less stimulating environment, and administering daily care for the individual, is of benefit for hyperthermia patients (38, 44, 88). Recently, a pilot study showed that a lower room temperature was associated with PSH, indicating that environmental interventions could complement pharmacological strategies (e.g., standardization of room temperature and application of a blanket) (89). A timely and accurately recorded monitoring index may assist the development of an appropriate therapeutic plan (65, 88). In this respect, frequency, duration, and severity, and the skills to mitigate the potential triggers of PSH, are thought to play important roles in non-pharmacological treatments (90).

Supportive therapy helps to improve long-term outcomes of PSH patients (3). A previous report suggests that energy consumption is increased up to three times the baseline during paroxysm, and caloric requirements are higher than might be expected for slow weight gain (41). Positive and professional energy setting that provides optimum nutrition may circumvent morbidity or reduce the long-term mortality of PSH (40). Currently, careful monitoring of nutrition, hydration, and mineral supplementation and early implementation of enteral feeding are important in nutrition management (49). Meanwhile, some issues such as full integration of individual nutrition and hydration requirements, tolerance of TBI patients during the course of PSH development, and the extent to which calorie intake can compensate for increased energy expenditure have emerged in the therapeutic setting.

Given that the occurrence of PSH may result from the presence of cerebral hypoxia, hyperbaric oxygen therapy (HBOT) in the management after TBI has been reported in a series of refractory PSH cases. Patients benefit from the increased oxygen availability due to improvements in cerebral aerobic metabolism in injured tissue (91, 92). The possible activation of the functions of devitalized neurons, and protecting undamaged nervous tissue through external intervention, will become additional means of PSH treatment. In addition, with the current trend for early rehabilitation after TBI that can improve prognosis of patients, researchers point out that physiotherapy, an important adjunct to pharmacological treatments, will extend the motion range and prevent contractures in PSH patient with posturing (3). Although the abovementioned therapeutic methods are used to decrease the frequency and duration of PSH, they are all symptom-oriented and consistently lack pragmatic proposals (14, 44, 48, 60, 93). Moreover, published reports, rather than recommendations for treatment, may be favored, and may not be uniformly suitable for clinicians with different levels of experience.

### An Algorithm for the Management of PSH in TBI Patients

**Figure 3:**
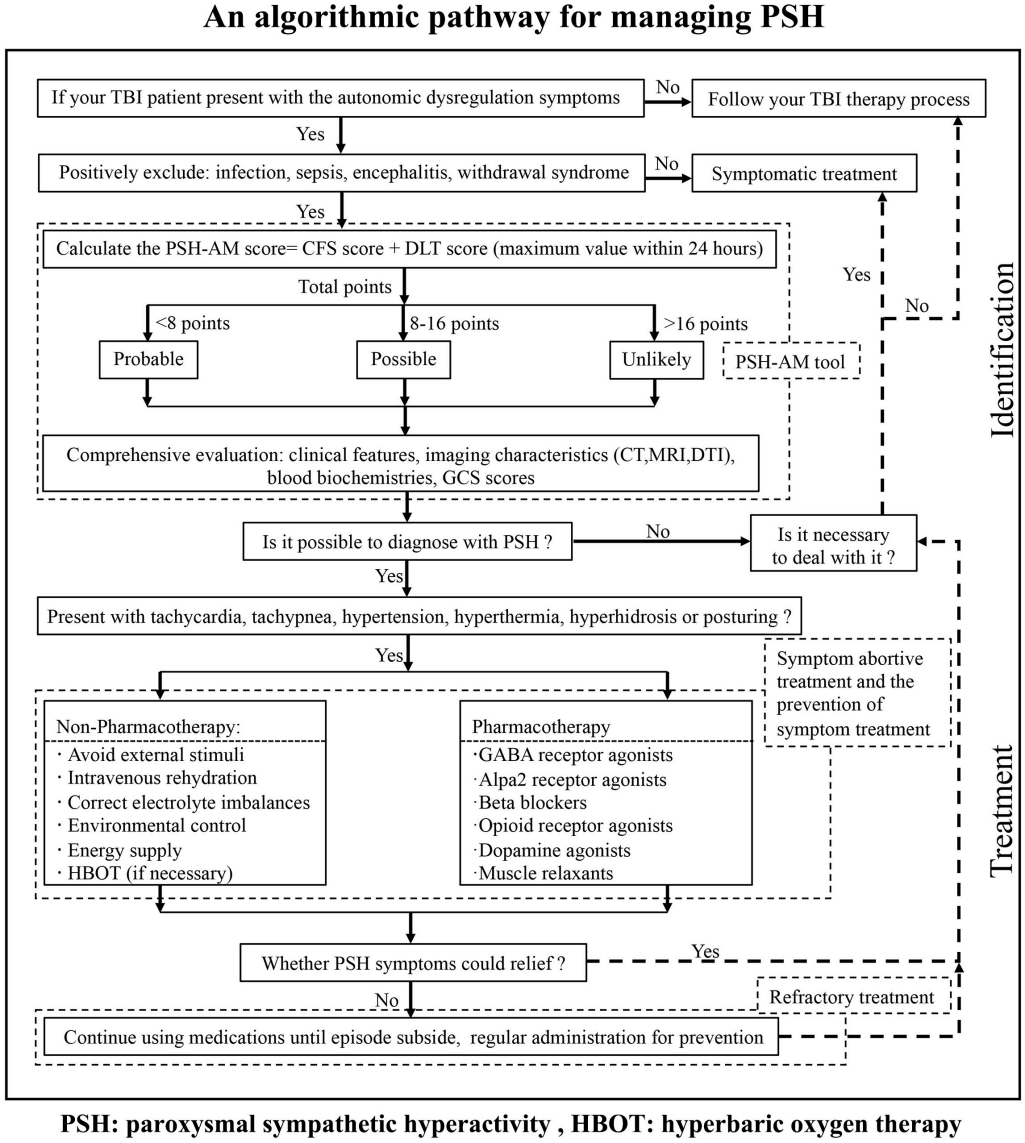
An algorithm for the management of PSH in TBI patients, which gives a brief pathway for the identification and management of PSH.

We integrated conventional references and then designed the protocol named “an algorithm for the management of PSH” (For details, see Figure 3) for the identification and management of PSH after TBI in our institution. The first step in starting the diagnosis is to make sure your patient has a clear history of head injury. The individual medical records including vital signs, nursing notes, and other clinical notes were reviewed to follow our algorithm. This institutional protocol exemplifies the pathway for the management of PSH in TBI patients.

Our primary motive was to decrease missed diagnosis and misdiagnosis, and further avoid excessive medication, to improve the prognosis of our TBI patients in our institution. Meanwhile, this algorithm will provide one standardization screening procedure to address the limitations in individual identification and management of PSH. Although our protocol is simple and has not been certified by experimental findings, it will enormously raise our general awareness, enhance different levels of diagnosis and treatment ability, and possibly promote the development of guidelines for PSH management in the future.

## Conclusion

It is generally known that PSH is mostly prevalent in TBI patients, and the understanding of advanced pathophysiological, clinical features, and recognition methods of PSH in TBI patients is necessary.

Although previous studies have not given a definite explanation, the disconnection theory and excitation:inhibitory ratio model were established foundations for the subsequently pathophysiological exploration. The findings of neuroendocrine disorders reveal a new sight of pathological analysis. Nowadays, more and more attention has been put in quantifying the frequency, severity, and duration of paroxysmal episodes. Given the complexity and diversity of PSH manifestations, early identification of the location and severity of TBI is essential for clarifying the neuropathology of PSH symptoms. Some features detected from the evidence-based clinical practice will provide predictors for early identification of PSH in TBI patients. Moreover, some risk factors such as age, early fever, GCS score, and the use of tracheostomy have been reported to be associated with the development of PSH. The development of laboratory examination and neuroimaging findings would provide more objective basis for the early diagnosis of PSH. The PSH-AM tool, which has been approved increasingly by clinicians, was the most optimum in early identification and prospectively detects the rate and stratifies the symptomatic severity of PSH by far. With the help of PSH-AM, a decline in the prevalence of PSH was revealed in TBI patients. In the future, rigorous investigations and prospective studies are needed to present more reliable data, for example, to establish whether individual management modulates the relationship between the severity of PSH and long neurological outcomes in TBI patients and to stratify complications caused by PSH as an entirety or PSH associated with the TBI.

The advances in treatment of PSH are collected from the relevant literature reports. However, compiling universal protocol, using uniformly evaluation criteria, and accumulating the multi-center medical reports to minimize data bias remain challenging. It remains to be seen whether we can co-opt the therapeutic experiences of acute TBI to ease PSH symptoms. On the basis of the therapeutic regime reported in previous case series and small trials, more scientifically and rationally designed clinical trials to judge the putative benefits of PSH are needed.

## Author Contributions

Z-QL and R-ZY contribute to literature extraction and prepared the manuscript. R-ZZ drafted this manuscript. G-HH and G-MZ supervised the study. All authors read and approved the final manuscript.

### Conflict of Interest

The authors declare that the research was conducted in the absence of any commercial or financial relationships that could be construed as a potential conflict of interest.
